# The effect of participatory community communication on HIV preventive behaviors among ethnic minority youth in central Vietnam

**DOI:** 10.1186/1471-2458-12-170

**Published:** 2012-03-08

**Authors:** Huy V Nguyen, Giang M Le, Son M Nguyen, Mai N Tran, Nguyet M Ha

**Affiliations:** 1 Institute for Preventive Medicine and Public Health, Hanoi Medical University, 01 Ton That Tung Str., Dong Da Dist., Hanoi, 10000, Vietnam; 2 Institute for Preventive Medicine and Public Health, Hanoi Medical University, 01 Ton That Tung Str., Dong Da Dist., Hanoi, 10000, Vietnam; 3 Institute for Preventive Medicine and Public Health, Hanoi Medical University, 01 Ton That Tung Str., Dong Da Dist., Hanoi, 10000, Vietnam; 4 Faculty of Nursing, Hanoi Medical University, 01 Ton That Tung Str., Dong Da Dist., Hanoi, 10000, Vietnam; 5 Vietnam Authority of HIV/AIDS Control, 135/3 Nui Truc, Ba Dinh Dist, Hanoi, 10000, Vietnam

**Keywords:** Ethnic minority youth, HIV/AIDS, Participatory community communication (PCC), Propensity score matching (PSM), Vietnam

## Abstract

**Background:**

In Vietnam, socially marginalized groups such as ethnic minorities in mountainous areas are often difficult to engage in HIV research and prevention programs. This intervention study aimed to estimate the effect of participatory community communication (PCC) on changing HIV preventive ideation and behavior among ethnic minority youth in a rural district from central Vietnam.

**Methods:**

In a cross-sectional survey after the PCC intervention, using a structured questionnaire, 800 ethnic minority youth were approached for face-to-face interviews. Propensity score matching (PSM) technique was then utilized to match these participants into two groups-intervention and control-for estimating the effect of the PCC.

**Results:**

HIV preventive knowledge and ideation tended to increase as the level of recall changed accordingly. The campaign had a significant indirect effect on condom use through its effect on ideation or perceptions. When intervention and control group statistically equivalently reached in terms of individual and social characteristics by PSM, proportions of displaying HIV preventive knowledge, ideation and condom use were significantly higher in intervention group than in matched control counterparts, accounting for net differences of 7.4%, 12.7% and 5%, respectively, and can be translated into the number of 210; 361 and 142 ethnic minority youth in the population.

**Conclusions:**

The study informs public health implications both theoretically and practically to guide effective HIV control programs for marginalized communities in resources-constrained settings like rural Vietnam and similar contexts of developing countries.

## Background

The overall picture of HIV/AIDS in Vietnam continues to be worrying over time. Since the first case of HIV infection sexually transmitted in 1990 [1], it then has spread dramatically through both risk sexual and drug use behavior across the country, with 160,019 cases reported nationwide [2]. To date researchers and policy makers remain uncertain about further development of this epidemic as well as progression of prevention efforts. Given previous research and literature, the HIV transmission has been focused largely upon injecting drug users (IDU) and female sex workers (FSW) due to their unsafe behaviors such as sharing injecting equipment and non condom sex [1,3-6]. However, much attention to such high-risk groups may restrict our understanding of other groups that may be also at risk of being affected by the epidemic [7,8]. Several subpopulations in Vietnam such as migrants, militants, and rural communities actually have also been believed as sufferers. Studies in some developed and developing countries have identified the association of migration [9-11], social contexts [12], and deficit in HIV prevention knowledge [8], social vulnerabilities of marginalized groups [13,14], and lack of access as well as insufficient and incomprehensive approaches to HIV preventive information and programs with increased epidemic [15].

Although fighting against the HIV/AIDS spread in Vietnam has become comparatively successful in several aspects, there are high risk groups that have not been engaged in HIV-related research and prevention programs. The recent data show that in Vietnam HIV cases have been reported in all 63 provinces and cities, almost 98% of districts, and more than 70% of wards, communes, and towns [2]. This means that the HIV epidemic has affected not only high-risk groups in urban areas, but other communities, especially ethnic minority youth in rural settings due to many individual and social factors [16].

In the Mekong river sub-region, the East-west Economic Corridor (EWEC) is one of the three routes with 1,600 km in length to connect the Indian and Pacific Oceans. The corridor has been built on the route that was known during the Vietnam War as "Route Number Nine" running through two mountainous districts, namely Dakrong and Huong Hoa, the very poor districts of Quang Tri province in central Vietnam. These districts have two main ethnic minorities Pahco and Vankieu, bordering with Laos through Lao Bao border gate. The completion of this route paved regional mobility and helped generate trade opportunities as well as facilitate cultural exchange for the region, especially between Laos, Thailand and Vietnam. The development of road infrastructure in rural residential areas has led to an increase in trade and accessibility and has therefore spurred economic and social development. Local people are increasingly able to access previously inaccessible services and trade opportunities. The development of routes, however, has influenced the social habits of affected communities in unexpected ways such as prostitution, drug use, sexual abuse and harassment and others. Many of these changes have the potential to negatively affect public health, especially by increasing unprotected groups' risks of contracting HIV and sexually transmitted diseases (STDs). In the previous baseline survey of the project "*Building a community-based pilot model for preventing HIV/AIDS in two mountainous districts of Quang Tri province"*, it was found that in parallel with the process of migration the facilitators and risk factors that increased transmitting HIV included changed lifestyles, social norms in favor of pre-marriage sex, local tradition "di sim" for seeking sex partners, non-condom sex due to limited perceptions of preventing HIV as well as lack of access to HIV prevention services [17]. Using the results of that survey, we implemented a variety of PCC that aimed to promote perceptional and behavioral change for preventing HIV among ethnic minority youth of some communes in Dakrong district of Quang Tri province.

In evaluating the impact of a communication campaign, program officers and researchers would like to be able to estimate the effect of intervention that is designed to change a behavior. It has been well recognized among researchers and evaluators that calculating effectiveness is the most important part, but is sometimes the most challenging [18]. One can not claim a particular amount of behavior change without a causal attribution. A causal inference must be reached that attributes the net change in behavior to exposure to the intervention and not to other impacts or, worse yet, to changes that occurred before the intervention was implemented. Because in this study we launched the intervention across the villages of two communes Dakrong and Ango and because residents including respondents were highly mobile between intervention and control villages, it is a difficult task for researchers to design a randomized control group study.

*The objective of this study was to estimate the relatively net effect of participatory community communications (PCC) on ideational and behavioral change for HIV prevention among ethnic minority youth in a mountainous district in central Vietnam using propensity score matching (PSM)*.

From literature PSM is highly recommended as it is one of the strong statistical techniques [18-20]. This method can help reduce selection bias as it allows for quasi-experimental contrasts between subjects receiving "treatment" and those in "control" groups based on their observed characteristics. Proper use of PSM should also allow for rigorously derived and relatively unbiased estimates of communication effects on participants' behavior [21]. Because of its ability to reduce selection bias, PSM has become increasingly used in the fields of education [22], communication [20], medicine and epidemiology [23], policy evaluation [24], economics [25], and psychology [26]. Although commonly applied in diverse disciplines in various settings, too little has been achieved in measuring the effect of PCC on HIV preventive behavior among ethnic minority youth in marginalized areas of developing countries like Vietnam.

**Figure 1:**
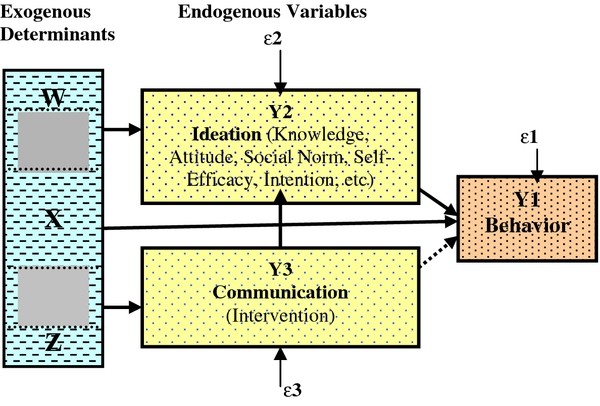
**A model of behavioral change communication **[**19**,**27**].

A theoretical framework for this study was based on a comprehensive conceptual model by Kincaid [19,20] (Figure 1) that has been adapted from a wide range of literature resources. Under this theory, the psychological influences including knowledge, attitude, social norm, intention, self-efficacy, and others can be combined as ideation. Specific communication interventions may be designed to influence only one or several types of psychological processes. All sorts of psychological processes are expected to affect behavior even if communication is designed to influence only one of them. Communication affects behavior indirectly by providing information that changes one or all of such processes. Exogenous determinants including demographic, socioeconomic and contextual characteristics affect endogenous variables such as recall of communication messages, ideation and behavior.

## Methods

### Study design and settings

A cross-sectional sample survey was conducted with after PCC intervention had been completed using a face-to-face structured questionnaire in some villages of Dakrong and Ango commune from Dakrong district in Quang Tri, central Vietnam.

### Intervention campaigns and settings

A multi-campaign intervention was designed and engaged with local communities, most of whom were ethnic minority youth in two communes-Ango and Dakrong-Dakrong district, Quang Tri province. Before the intervention, a group of the local ethnic minority youth was recruited to participate in intervention. They were encouraged to visit local households and attend social events at their home villages to collect local stories that reflected messages on HIV/AIDS prevention, then discussed with research team to choose the relevant messages. In final analysis, there were nine key messages that conveyed the contents on HIV/AIDS prevention including 1) *Be faithful with one wife and one husband*, 2) *Practice safe sex*, 3) *Don't have sex with sex workers*, 4) *Use condoms correctly when having sex*, 5) *Be friendly with condoms*, 6) *Don't inject drugs*, 7) *Don't share syringes and needles*, 8) *Take a test for HIV*, and 9) *Care for pregnant women*.

In total, there were also nine communication campaigns, each of which incorporated several or all of such messages in order to promote HIV/AIDS preventive behaviors among ethnic minority youth. The first campaign "*HIV/AIDS prevention drama*" was launched from October 2009 to July 2010. Using local stories, 38 ethnic minority youths involved in drama technically guided and supported by a local director. The second campaign "*Women's health clubs*" aimed to encourage open dialogues and interactive talks with ethnic minority women on HIV prevention topics from October 2009 to July 2010. By April 2009, "*HIV/AIDS knowledge contest*" was carried out with the participation of close to 1000 ethnic minority youth across two communes. Another competition with a topic "*Be friendly with condoms*" took place in December 2009 and there were 600 ethnic minority youth engaged in this campaign. Within the July 2010, the next contest named "*Typical role models*" attracted 900 ethnic minority youth involved. Meetings by 400 ethnic minority youth and the live show by 1600 ethnic minority youth were held for promoting "*Be friendly with condoms*" during the December of 2009. During this time, community-based clinics were also established for providing examinations and consultations on STD prevention and treatment for more than 1800 local women at reproductive age. A total of 64 community initiatives for preventing HIV/AIDS, each of which mobilized 70 to 100 ethnic minority youth involved, were finally successfully designed and promoted by such ethnic minority youth themselves from October 2009 to July 2010.

### Participants and sample size

A representative sample survey of 800 ethnic minority youth aged 15-45 years from 2 communes-Ango and Dakrong-in Dakrong district, Quang Tri province were selected and interviewed by August 2010. This sample size was obtained by the cluster sampling with identified 40 random clusters. A cluster is defined as a number of households or families living fairly close to one another in a group of residents in each village. To increase the representativeness, villages with more populations had higher chance to be included into the sample.

### Data collection

As a procedure, this intervention project was approved by the Human Research Ethics Committees at the Hanoi Medical University. After the intervention, participants in the survey were verbally informed about the study, that participation was voluntary, that they had the right to withdraw at any point, and, that data would be handled confidentially. After obtaining informed consent, a structured questionnaire was administered to participants on a face-to-face basis of interview. The survey was anonymous without documenting participants' names in any research materials. The survey was conducted by well trained interviewers-both men and women to conduct gender-matched interviews (men vs men and women with women). These interviewers were community health workers who were experienced with the face-to-face interviews in many community research surveys before. Households that stayed close about 10 metres in distance to each other were identified as a cluster. The first household interviewed was randomly selected. When data collectors entered a household, they interviewed all youth aged 15-45 years, thereafter moved to another until they reached roughly 20 youth in each cluster and 800 participants in total in a door-to-door approach.

### Measurement

#### Exogenous variables

The socio-demographic characteristics were measured with age (number of years), gender (male, female), ethnic (Pahco, Vankieu), marital status (married, unmarried), mobility (migrant, non-migrant), socioeconomic status [SES] (a composite of education level, Kinh language competency-listening, speaking, reading and writing-and number of valuable properties, Cronbach's alpha = .65) with higher scores reflecting higher SES, social network (a composite of number of friends and relatives in other areas, number of visits to relatives during the past three months, distance to the closest friend or relative, and joining local social organizations (Cronbach's alpha = .55), and access to HIV/AIDS preventive information. Access to HIV/AIDS preventive information was assessed with 7 items regarding sources of information accessed, of STDS listed, of local projects and programs known, of AIDS prevention activities of our project known, of times watching or joining AIDS preventive dramas and of times joining community initiatives for preventing HIV (Cronbach's alpha = .85); the sum scores with higher scores reflect higher level of access to HIV/AIDS preventive information.

#### Recall

Actual exposure to PCC for HIV prevention may be measured as a state whether a youth has participated in an intervention of any kind. However, a good method of communication evaluation requires a valid, reliable measure of recall of key messages or contents at the interval level of measurement [18,27]. In this study, a category-coded question was added to ask students to remember all possible communication messages. In total, 9 key messages were recalled by participants. This question formed a continuous scale measuring the level of recall that ranged from 0 to 9 messages (Cronbach's alpha = .80). To simplify this measurement and accommodate the logistic regression and propensity score analysis, the scale was classified into 1 and 0 (recall versus no recall).

#### Ideation

HIV preventive knowledge was assessed with 15 true/false/don't know items adapted from the previous measures [28,29]. Scoring the information scale was accomplished by dichotomizing each item into a value of 1 (correct) and 0 (incorrect or don't know) and then summing the item values to form a composite score with higher scores reflecting increased knowledge about HIV prevention (Cronbach's alpha = .83).

To determine respondents' attitude toward HIV prevention such as condom use, respondents rated their performance of 4 prevention acts on a 2-point semantic scale (agree and disagree) from 0 (negative evaluation) to 1 (positive evaluation) [e.g., "*How good or bad would it be if you talked about condom use (to keep from getting HIV/AIDS) with your sex partner(s) before having sex with them*?"] [29]. A composite score was obtained by summing responses to items with higher scores indicating higher levels of attitude toward HIV prevention (Cronbach's alpha = .75).

Social norms assesses respondents' subjective norms of social support for their HIV prevention practice (e.g., "*Do people in your village think you should talk about condom use with your partner(s) before having sex with them*?") [29]. A composite score was retained by summing responses to 5 yes/no items on this scale with higher scores indicating higher levels of social norms toward HIV prevention (Cronbach's alpha = .40).

Perceived risk of HIV infection consisted of 3 yes/no items asking if respondents thought they were at high risk of HIV infection (e.g., "*Are you worried at risk of HIV infection if you had sex with commercial sex workers*?") [29]. A composite score was computed by summing responses to items with higher scores indicating higher levels of perceived risk (Cronbach's alpha = .78).

Self-efficacy of HIV prevention was assessed with 4 yes/no items tapping perceived difficulty of HIV prevention practice such as condom use on the scale from *hard *(0) to *easy *(1) such as "*how hard would it be for you to advice or persuade your sex partners to use a condom when having sex with them?*" [29]. A composite score was obtained by summing responses to items with higher scores reflecting higher levels of self-efficacy (Cronbach's alpha = .76).

These five related sub-constructs representing the cognitive and social interaction component of ideation were used to construct the latent measure of ideation for HIV prevention (Cronbach's alpha = .77). For the logistic regression analysis, using a cut-off of 50% the measures were split into 1 and 0, corresponding to higher and lower levels of ideation.

#### HIV prevention behavior or condom use

Protected sexual behavior combined 3 items asking if participants used condoms at the last sex with sex partner(s), if they used condoms for HIV prevention or other purposes and if they used condoms before or after insertive sex. Combining these 3 items into a single outcome variable has two advantages in that it makes the measurement more valid and reliable as well as allows the analysis of the impact of intervention [18,27]. The levels of protected sexual behavior met the minimal requirements of order with respect to ideation with the support from one-sided test of significance [30] performed with *P *= .01. This level of probability indicated the rejection of the null hypothesis of equality of levels and supporting the alternative hypothesis of order. To make logistic regression analysis and estimation of the impact possible, the scale of the single sexual behavior was classified as 1 and 0 reflecting safer and riskier level of sexual behavior (Cronbach's alpha = .67).

### Data analysis

#### Trend and Jonckheere's one-sided test

These statistics tested if there is an increased trend according to the level of exposure (recall) to the intervention.

#### Simple proportion differences

We used Chi-square tests to determine whether proportion differences in knowledge, ideation and condom use of students receiving and not receiving PCC were statistically significant. We used a *P *value of .05 for these analyses. We considered the results obtained from Chi-square tests of these proportion differences as a "benchmark" for the further analyses using PSM as referred to below.

#### Logistic regression modeling

The model of direct and indirect effects used for this analysis requires three equations, one for each endogenous variable:

$$\begin{array}{c} {\text{Y}}_{1\text{i}}=\beta_{0}+\beta_{1}{\text{Y}}_{2\text{i}}+\beta_{2}{\text{Y}}_{3\text{i}}+\beta_{3}{\text{X}}_{\text{i}}+\mu_{\text{i}}\\ \;{\text{Y}}_{2\text{i}}=\gamma_{0}+\gamma_{1}{\text{Y}}_{3\text{i}}+\gamma_{2}\;{\text{W}}_{\text{i}}+{\text{v}}_{\text{i}}\\ {\text{Y}}_{3\text{i}}=\sigma_{0}+\sigma_{1}\;{\text{Z}}_{\text{i}}+\xi_{\text{i}} \end{array}$$

Where Y_1i _is safer sexual behavior for subject i, Y_2i _is ideation, Y_3i _represents exposure to communication for subject i, X_i_, Wi and Zi are matrices of exogenous socioeconomic and demographic control variables, the three β, two γ and one σ coefficients are parameters to be estimated from the data, and μ_i_, vi, and ξi are the disturbance (residual) terms. Because safer sexual behavior, ideation, and exposure are measured with a binary scale, logistic regression is used to estimate the parameters of the equation. The differentiation among the X, Z, and W matrix of exogenous control variables indicates that each endogenous variable should be determined by exogenous variables not included in the other two equations. However, some overlap of exogenous variables can be acceptable, but each endogenous variable must have at least one exogenous control variable that is excluded from the equations for all other endogenous variables [31].

#### Creating the matched sample using propensity score matching

A propensity score is the probability of being exposed to a treatment or an intervention given a set of observed covariates, *X*. The method as this was developed as a means to balance the treatment and control units so that a direct comparison would make a valid conclusion. The technique was found robust as recently in practice it was difficult, if not possible, to match on more than two variables unless PSM is used [32,33]. For research survey, a single score for matching is generated using statistically regressing exposure on all of the variables that determine exposure and also may be related to the outcome variable [18].

This technique requires a two-stage process. Stage 1 involves the use of a logistic or probit regression model to calculate all respondents' propensity for experiencing a treatment of interest, in this case, receiving PCC. In stage 2, we used the estimated propensity scores obtained in stage 1 to match youth who did and did not receive PCC. To obtain a full sample, we used stratification matching which uses all treatment and control cases. Using the STATA 10.0, the full range of sample members' propensity scores is divided into propensity score strata, or blocks, each of which includes treatment and control cases with the same or nearly the same propensities for receiving the treatment. The number of appropriate strata depends on the number necessary to gain a balanced propensity score. Within each of these strata, the ATT (Average Treatment Effect for the Treated) is calculated, and then the ATT's across strata are averaged to produce a final ATT.

## Results

### Socio-demographic characteristics of the sample

The distribution by gender (male vs. female) and marital status (married vs. unmarried) was relatively equal, with average age of 24 years old. Most were ethnic people-Pahco (41.43%) and Van Kieu (56.32%), few were Kinh people (2.3%). In terms of SES, all indicators reflected the lower value: incomplete secondary education (close to grade 6), most respondents were able to speak and understand Kinh language (> 95%), but only 71% were fluent in reading and writing this language. The most valuable asset was television (70%), followed by motorbike and mobile phone, each making up more than 40%. The SES score was around of mid point (mean = 5.45, range = 0-11). The temporary migration percentage was rather high (almost 50%), whereas the level of social connection was low (mean = 2, range = 0-6). Generally, access to HIV/AIDS information from different sources was low (only 24% had good access); among the communication messages, the three messages "*Use condoms correctly when having sex*", "*Practice safe sex*" and "*Be faithful with one wife and one husband*" were recalled the most (17%, 11% and 9%, respectively). The overall rate of recall of at least one HIV prevention message was more than one-fifth (22.1%).

### Simple changes and differences

**Figure 2:**
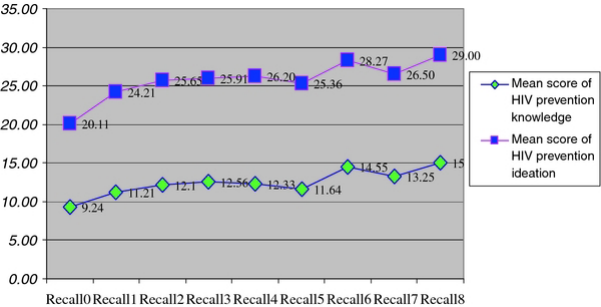
**HIV prevention knowledge and ideation by level of recall of HIV prevention messages (*P*-value = .000, Trend test and Jonckheer's one-sided test)**.

Figure 2 shows the increasing trend in the mean scores of HIV prevention knowledge and ideation according to the extent which participants recalled communication messages of HIV prevention (*P *< .001).

Table 1 shows that the percentages of knowledge and ideation were significantly higher in recall over the non-recall group (*P *< .001), but the difference in condom use prevalence was not significant.

**Table 1:** Unadjusted differences in HIV preventive knowledge, ideation and behavior between exposed and non-exposed youths

Variables	Exposed group (recall) n (%)	Non exposed group (not recall) n (%)	*P *(χ2)
HIV preventive knowledge	157(88.70)	342(54.90)	***
HIV preventive ideation	169(95.48)	373(59.87)	***
Safer sexual behavior (condom use)	70(39.55)	229(36.76)	NS

*P < .05; **P < .01: ***P < .001; NS = Not significant

### Logistic regression modeling for predictors of recall, ideation and APB

The second column in Table 2 is the descriptive statistics of variables and factors of each model. The models of recall of HIV preventive communication messages, ideation and behavior for HIV prevention were in turn identified by six factors each (*P *of most factors < .05). The results of the statistical test to exclude inappropriate variables and factors from each model are presented in the second row from the bottom. In all models, the exclusion of some factors did not change the parameters of each model (*P *> .05). HIV preventive ideation, after controlling for socio-demographic variables, was the most significant predictor of condom use behavior (OR = 2.38; *P *< .001). The results suggested that recall of HIV prevention messages has direct impact (*P *< .05) on ideation, but has indirect impact on condom use (*P *> .05). Analysis of the "biprobit" equation shows that the correlation coefficient "rho" between HIV preventive ideation and condom use behavior was very low, especially since "correlation errors" were not significant (*P *> .05).

**Table 2:** Regression modeling for factors affecting HIV preventive behavior

Variables	Mean (Range)	%	Recall of intervention messages OR(CI)	HIV preventive ideation OR(CI)	Safer sexual behavior OR(CI)
Age (higher vs lower)	24.17 (15-45)		0.96* (0.92-0.99)	0.97* (.95-0.99)	1.04* (1.01-1.06)
Gender (male vs female)		52.63	_	_	1.79*** (1.27-2.52)
Ethnic (Vankieu vs Pahco)		57.62	0.48** (0.30-0.78)	0.57** (0.39-0.81)	_
Marital status (married vs unmarried)		55.25	_	_	6.75*** (4.36-10.43)
SES (higher vs lower)	5.45 (0-11)		0.95 (0.84-1.09)	1.20*** (1.09-1.31)	_
Migration (yes vs no)		49.88	0.65 (0.37-1.12)	_	_
Social network (higher vs lower)	2.00 (0-6)		1.51*** (1.24-1.44)	1.21** (1.06-1.37)	_
Access to HIV information (higher vs lower)		24.00	1.37*** (1.31-1.44)	1.15*** (1.09-1.22)	1.05** (1.01-1.08)
Exposure to intervention (recall vs non recall)		22.13		3.24** (1.38-7.56)	0.67 (0.40-1.23)
HIV preventive ideation (higher vs lower)		67.75			2.38*** (1.62-3.51)
Sample	800	800	800	800	800
Exclusion test (model fitness): χ^2^(*p*)^1^				1.95 (0.16)	0.04 (0.84)
Test for endogeneity: rho (CI)				0.49 (-.24-0.87)	-0.04 (-.46-0.39)

(-) = variables excluded from the model in order to increase the level of statistical significance of the parameters and model fit

^1^Likelihood test applied for binominal variables

*P < 0.05; **P < 0.01; ***P < 0.001; NS = not significant

### Propensity score analysis

Table 3 presents the results underlying propensity score matching (PSM) analysis according to the stratified probability score methodology, aiming to maximize matching all observed individuals (n = 800) to obtain the intervention group and control group statistically balanced in terms of socio-demographic characteristics. Propensity score is the probability of recalling HIV preventive communication messages of the intervention, with mean of 0.21 (range = 0.001-0.999; SD = 0.27; data not shown in the interest of space). Such propensity scores were stratified into 6 strata or 6 blocks by which external factors or confounders of the intervention group and the control group, including age, ethnic, SES, social network, migration and access to information were equivalent (6 strata and 6 exogenous factors meaning 36 separate tests which showed statistically no significance) [Data not shown in the interest of space]. The results, after being adjusted by PSM, showed that there were statistically significant differences [*P *< .05] between intervention group and control group in proportions of knowledge, ideation and condom use. Participants who recalled HIV preventive communication messages were more likely to have better knowledge, ideation and behavior for HIV prevention as compared to those who did not recall any messages, specifically knowledge (7.4% higher), ideation (12.7% higher), and safer sexual behavior (5.0% higher) [*P of *Z-test < 0.05].

**Table 3:** The net differences in the percentage of HIV preventive ideation and behavior (condom use) between the two groups adjusted by PSM

Outcome variables	Number of blocks balance#	Non exposed group (A) N	Exposed group (B) N	Total	Net difference = B-A using "Atts" command %	Converting percentages into numbers
HIV preventive knowledge	6	623	117	800	7.4%**	210
HIV preventive ideation	6	623	117	800	12.7%**	361
Safer sex (condom use)	6	623	117	800	5.0%*	142

*# *indicating the number of balanced blocks in terms of characteristics or statistically insignificant difference in characteristics between non exposed group and exposed group, meaning that the propensity score was balanced when stratified into 6 blocks, which allow for calculating the net impact of the intervention as compared between the two groups. *P < 0.05; **P < 0.01; ***P < 0.001

**Figure 3:**
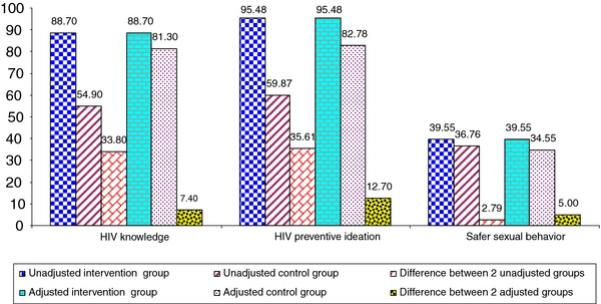
**Comparison of the unadjusted increase in HIV preventive knowledge, ideation and condom use to the increase adjusted by PSM (N = 800)**.

Figure 3 presents the simple bar graph comparing the results of the analysis between being unadjusted and adjusted by PSM. The unadjusted differences in HIV preventive knowledge, ideation and condom use behavior between the treatment group and matched control group was 33.80; 35.61, and 2.80 percentage points, respectively. The adjusted differences between the treatment group and matched control group were 7.40; 12.70 and 5.0 percentage points. The estimated percentage point increase in knowledge, ideation and condom use as the impact of the PCC can be translated into the numbers of youth displaying knowledge, ideation and condom use by 210; 361 and 142 youth, respectively in the population (this calculation was based on the total population of 2844 ethnic minority youth). These numbers of participants demonstrating HIV preventive knowledge, ideation and condom use can be attributed to a recall of the communication messages related to HIV prevention.

## Discussions and conclusions

In this community-based intervention study, we sought to estimate the effects of launching communication campaigns for HIV prevention with the participation of local communities on increasing ethnic minority youth's HIV prevention behaviors. We did so using a fairly large sample of such youths and methods that can greatly reduce selection bias. Because we could not randomly assign local youth to receive or not receive communication messages, we used PSM techniques to contrast the HIV preventive behaviors of ethnic minority youth who did and who did not receive these campaigns but who had been matched on a wide range of observed background characteristics. We also conducted benchmark analyses using *trend *test, *Chi^2 ^*tests of proportion differences and logistic regression before further examination using PSM analysis.

### Evidence of the effect of participatory community communication

Our analyses indicate that the PCC campaigns provided to Vietnamese ethnic minority youth during the almost one year would be of sufficient strength to prevent risk behaviors for HIV infection. We found that PCC had positive effects on ethnic minority youth's HIV preventive behaviors. Youth displayed increased levels of knowledge and ideation for HIV prevention as they had increased levels of exposure to HIV preventive messages. These results are consistent with data in Kincaid's research [27] indicating that the levels of contraceptive knowledge and ideation among the Philippine people increased according to the increased levels of recall of communication messages, and contraceptive behaviors such as condom use among the Philippine people increased as knowledge and ideation with this regard increased. Other research in Africa also showed a similar pattern [34] as our study. In contrasting the proportion differences, we found that youth receiving PCC campaigns demonstrated significantly higher knowledge and ideation than closely matched peers not receiving such campaigns. Youth displayed statistically different knowledge and ideation gain between the two groups, in which exposed group displayed higher proportions than the other group. Likewise, exposed (or recall) group also showed a higher proportion of condom use than non-exposed counterparts. However, these two groups of youth had statistically equivalent condom use proportion. Interpretations of this lack of statistically significant effects on condom use would be problematic if we are based only on the simple statistical devices such as Chi square test for contrasting this effect. According to Morgan [35], using univariate analysis to interpret effects of intervention may be of great bias.

To further examine the effects of PCC, we continued to analyse data using multivariate regression procedure. After adjusting for socio-demographic variables, we found that youth who were more exposed to HIV preventive information were more likely to recall HIV preventive messages (OR = 1.37; *P *< .001). Youth who approached HIV preventive information and recalled communication messages were more likely to display a higher proportion of HIV preventive ideation (OR = 3.24; *P *< .01). Youth who displayed a higher proportion of HIV preventive ideation were more likely to engage in condom use when having sex with a sexual partner (OR = 2.38; *P *< .001). However, recall of HIV preventive messages was statistically not associated with condom use. These results indicated that recall indirectly affected condom use through ideation or put in other words ideation played as intervening or mediating role between exposure to intervention (recall) and behavior. This appears evident that PCC campaigns have changed HIV preventive knowledge and ideation of the youth first, and then influenced their behaviors. This result is in consort with data by Kincaid [27] and Kincaid and Do [18] suggesting that interventions have indirect impact on a HIV preventive behavior only as mediated by their impact on ideation.

The above results and discussions show the significant effects of the PCC on a number of outcomes such as knowledge, ideation and condom use behavior among the ethnic minority youth. However, these youth have not yet been matched on their propensity to receive and not to receive the communication campaign; as a result, such estimates may remain biased [18-20,27]. From these benchmark results, we continued to estimate the effects of the intervention based on the PSM as shown in Table 3. The results indicate significant differences in proportions of youth displaying HIV preventive knowledge, ideation and behavior (condom use) between youth who exposed and recalled and who did not expose and recall communication messages but who have been matched on their propensity to expose and recall HIV preventive messages. The combination of two main analyses-unadjusted estimates (trend, Chi square) and adjusted estimates (multivariate regression, PSM)-has made the justification of the net effects of intervention possible. The three equations (represented in Table 2) controlled for potentially confounding (socio-demographic) variables that might affect behavior. After controlling for these variables, the recall of HIV preventive messages had a significant effect on knowledge, ideation and behavior. The potential effect of unobserved variables (not in the equations) and the reciprocal effect of behavior on ideation and recall were ruled out by the statistical tests for endogeneity. The only criterion missing for a causal inference was a counterfactual condition which could have been provided only in a controlled experimental design. In our study, the counterfactual condition was made by PSM technique in order to create a matched controlled group so the comparison of net difference would be possible. However, acceptance of such a causal inference for recall and ideation on condom use does not necessarily mean that other causes were not also operating. Youth may approach or be exposed to other sources of HIV prevention such as internet or other projects. But at least in this study we argue that the effect on knowledge, ideation and condom use would be as a result of the PCC per se which was designed and delivered by our interventions because we measured the recall of the key contents provided by such campaigns. Comparing between the treatment group and matched control group, a percentage increase of 7.4, 12.7 and 5 in HIV preventive knowledge, ideation and condom use behavior, respectively, after the communication messages delivered through a variety of community-based communication campaigns may sound small. However, because the sample of 800 represents a population of 2,844 ethnic minority youth, the actual net increase in the number of youth displaying knowledge, ideation and condom use for HIV prevention is estimated to be 210; 361 and 142, respectively. The above proportions of preventive change are in mid-range as compared with those of contraceptives and condom use when having sex among the married spouses in the Philippines [18,27] as well as behaviors of condom use and HIV testing among the residents in Africa [34].

In this study, we did these estimates of PCC's effectiveness using rigorous methodology. We used multiple items to measure outcomes of interest such as HIV preventive knowledge, ideation and behavior. Each of these measures displayed comparatively strong psychometric properties. We also combined both unadjusted and adjusted analytical methods, especially PSM in estimating campaigns' impact. Our sample was selected from a fairly large-scale representative sample of Vietnamese ethnic minority youth. Collectively, these methods yielded the same general pattern of findings, which helps ensure that flawed methodology is an unlikely explanation for the study's findings.

### Limitations

Our study has several limitations. First, the propensity score model for recall of HIV preventive messages includes variables or factors, many of which were identified in prior research as predictive of the receipt of communication campaigns. However, our model may not have incorporated additional variables that predict a youth's receipt of such campaigns. Therefore, the results may be biased by hidden bias or omitted variables. However, we did not find any evidence of this bias based on our analyses. The test for endogeneity for indirectly assessing if there were any variables not included that affected the models shows a little value of rho coefficient (*P *> .05), suggesting there was no or little hidden bias.

The research is also affected by the limitations that apply to using self-report measures for sensitive issues such as sexual behavior. Recall and reporting bias that may give a rise to under-estimation would be inherent. However, as this study designed a survey with the anonymous and confidential commitment, it was expected to partly reduce such a bias. We conducted an intervention study using a not really longitudinal design because we launched interventions first, but only thereafter carried out a post-intervention sample survey; therefore, this cross-sectional design may limit the order of causality.

Our study was designed to provide a general or overall estimate of PCC's effects. Our intent-to-treat analyses provide estimates of PCC's "use effectiveness" rather than its "method effectiveness". We currently can not offer detail on the effectiveness of specific types of behavioral change communication in rural and remote settings. For example, we are unable to say whether participatory community intervention, as we did in the current study, was more or less effective than non-participatory approach. Further, our point estimates of communication campaigns' effects are limited to ethnic minority youth in central Vietnam, therefore, may not be generalized across the country.

## Conclusions and implications of the study results

This study, to our knowledge, is the first to examine the effects of community-based intervention campaigns for HIV prevention applied to ethnic minority youth. Interpretations of this study provide public health implications both theoretically and practically. Consistent with the literature, our study supports the indirect effect of the communication (message recall) on behavior or the intervening role of ideation between communication and behavior as shown in the theoretical framework. This suggests that designs of intervention and evaluation should include ideation in addition to the communication in order to obtain a holistic theoretical model to support research. Communication interventions should design campaigns that help maximize actual exposure such as recall of communication messages and improve knowledge and ideation so the campaigns are more likely to increase practices of healthy behavior. This study therefore hopefully will lead to an increased understanding of how to improve perceptions and change behaviors for preventing HIV in ethnic minority young communities.

Using the PCC as an intervention approach, this research helps to empower local people address their own problems by themselves. We involved local ethnic minority people and youth to identify their own health problems, develop their local stories for dramas and participate in a number of activities such as HIV preventive dramas, open dialogues, knowledge contests, meetings and health check and treatment for STDs, and others. We believed that knowledge and skills obtained from the current interventions help ethnic minority youth continue to address their unaddressed health problems such as risky behaviors for HIV as well as other problems even though our interventions will not continue. The study therefore has made a significant contribution to achieving sustained results and outcomes. It is recommended that the model of PCC be rolled out to other rural areas in Vietnam and maybe to similar contexts of developing countries.

Given the limitations of the current study, it is recommended that future research consider a longitudinal design in other parts of the country such as southern and northern Vietnam in order to support temporal and generalizational inferences. Future research may also seek to compare and contrast the impact of PCC in relation to that of traditional approaches. In doing so, there are still more substantial opportunities to prevent the spread of HIV/AIDS among ethnic minority youth and other population groups, depending on the commitment, determination, and effort of researchers, policy makers and practitioners.

## Abbreviations

ATT: Average treatment effect for the treated; FSW: Female sex worker; IDU: Injecting drug user; PCC: Participatory community campaign; PSM: Propensity score matching; SES: Socioeconomic status; STD: Sexually transmitted diseases; WEEC: West-east economic corridor.

## Competing interests

The authors declare that they have no competing interests.

## Authors' contributions

HVN designed the intervention, designed the survey, trained data collectors, analysed data, drafted and revised the manuscript. GML designed the intervention project, designed the survey, and reviewed the manuscript. SMN designed the intervention project and the survey. MNT designed and supervised the survey. NMH designed the survey. All authors read and approved the final manuscript.

## Pre-publication history

The pre-publication history for this paper can be accessed here:

http://www.biomedcentral.com/1471-2458/12/170/prepub
